# Basal Cell Carcinoma of the 2nd Digit Treated With the Reverse Cross-Finger Flap: A Case Report

**DOI:** 10.7759/cureus.65105

**Published:** 2024-07-22

**Authors:** Arman Tabarestani, Anton Khlopas, Thomas Wright, Jongmin Kim

**Affiliations:** 1 Division of Hand and Upper Extremity Surgery, University of Florida, Gainesville, USA

**Keywords:** tumor, index finger, finger flap, basal cell carcinoma, aesthetic repair

## Abstract

Basal cell carcinoma (BCC) is the most common form of skin cancer but rarely presents on the finger with few cases described in the literature. We present the case of a 77-year-old female with a nine-month history of biopsy-proven BCC on the dorsal aspect of her left index finger. Following the complete surgical excision of the tumor, a two-stage reverse cross-finger flap was performed, resulting in minimal impact on mobility and aesthetics and prompting a discussion on the efficacy of this approach in treating BCC of the hand. We explore the various treatment modalities available for BCCs, underscoring the importance of tailored interventions for optimal patient outcomes. The successful outcome in our case emphasizes the significance of considering alternative surgical techniques in managing uncommon presentations of BCCs, contributing to the evolving armamentarium of options available to hand surgeons.

## Introduction

Basal cell carcinoma (BCC) is a slow-growing tumor of the skin and notably the most common malignancy in the United States [1]. The prevalence of BCC has increased in the last 25 years, partly due to the aging demographics but also because of the changing behavior of the population that increases the risk of exposure such as sunbathing or traveling [1]. BCC rarely metastasizes and thus has low mortality rates, especially if treated early in its clinical course. Common risk factors include previous sun exposure, light skin complexity, and immunosuppression [2]. Of note, the hand is subject to intense sun exposure but the occurrence of BCC in the hand is relatively uncommon [1].

Generally, the standard treatment for BCC is surgical excision, with Mohs micrographic surgery (MMS), electrodissection and curettage, cryosurgery, topical imiquimod, and topical fluorouracil also commonly employed in clinical practice [3]. Last resort treatment for appendages with BCC includes amputation due to delayed diagnosis.

Soft tissue defects in the hand arise from a multitude of different factors including trauma, infection, and in this case, tumor resection. While primary intention closure can be used for smaller defects, hand surgeons frequently employ local and regional flaps for larger lesions that are more exposed and involve the tendon, deeper muscular layer, or bone [4]. In this case, we performed a reverse cross-finger flap, which is designed to treat dorsal defects usually over the middle and proximal phalanges. This technique allows the transfer of subcutaneous tissue and vascular structures from the donor digit onto the defect of the recipient site that would not be as readily available with skin grafts. The patient was informed that data concerning the case would be submitted for publication and provided consent.

## Case presentation

**Figure 1:**
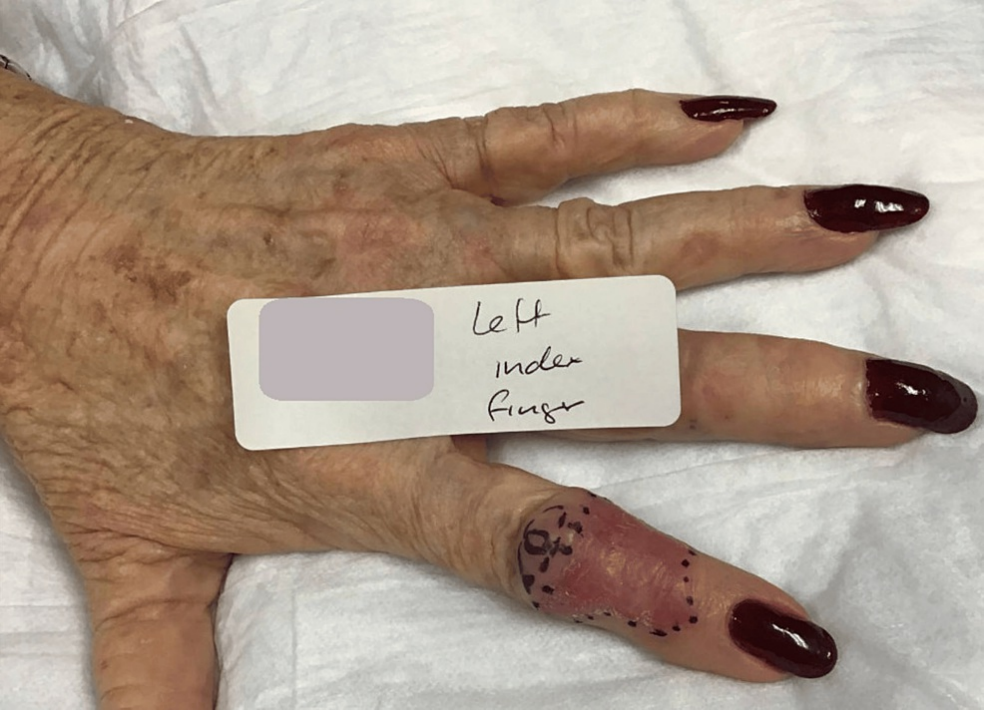
Preoperative image displays the BCC located on the index finger of the left hand. BCC: Basal cell carcinoma

A 77-year-old female presented with a four-month biopsy-proven ulcerated nodular type BCC on the dorsal side of the left index finger. It measured 3.4 x 2.3 cm and was characterized as a pearly, thin plaque. Additionally, she developed a 1.5 x 1.0 cm skin-colored nodule adjacent to the BCC and the proximal interphalangeal (PIP) joint (Figure 1). The patient denied any tenderness to palpation and had full motion of the PIP and distal interphalangeal (DIP) joints without pain. The plaque and nodule were mobile masses overlying the phalanges. 

The patient initially presented to Dermatology who performed a biopsy to determine if the nodule was part of the BCC or a separate entity. The biopsy confirmed that the nodule was consistent with BCC. An MRI of the digit was performed which demonstrated a very close association of the BCC with the dorsal hood. The patient was then informed by Dermatology she might need a finger amputation, which she was strongly opposed to. As a result, she presented to Orthopedics for further evaluation. We confirmed the diagnosis of BCC of the left index finger and planned for wide excision followed by full full-thickness skin graft or reverse cross-finger flap.

**Figure 2:**
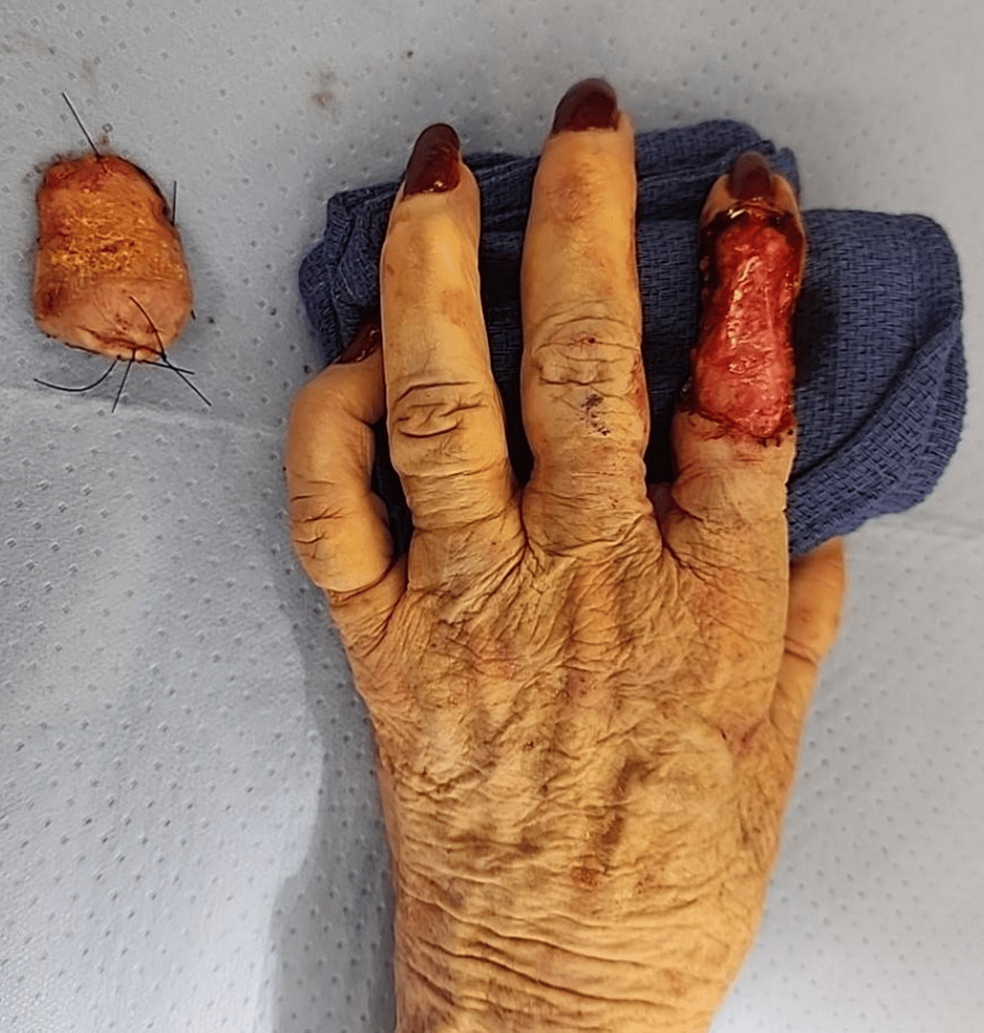
Intraoperative removal of the tumor before performing reverse cross-finger flap.

**Figure 3:**
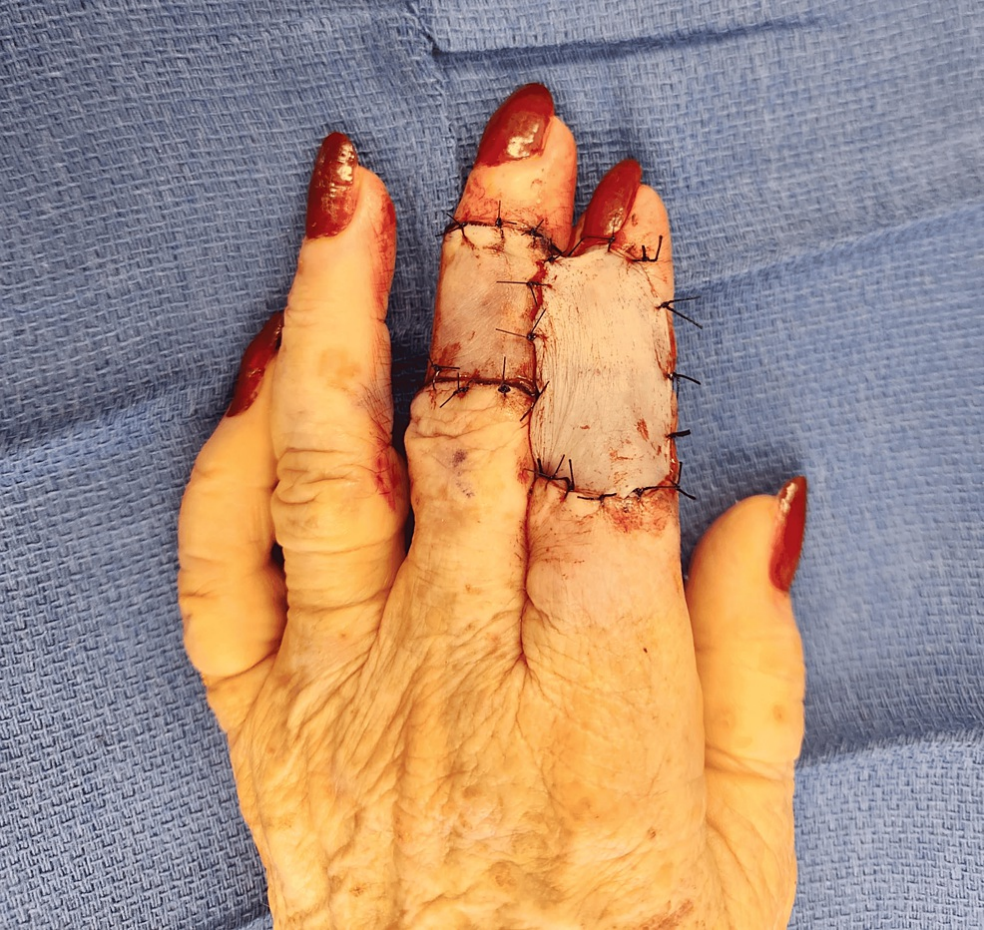
The 2nd and 3rd digits were joined upon completion of the reverse cross-finger flap.

During the operation, a 5mm margin of the tumor was excised as well as the entire nodule (Figure 2). We excised the tumor above the extensor tendon which was preserved at first. The frozen section result indicated that the deep margin was not clear. Thus, we proceeded to excise the extensor tendon. Afterward, we performed a reverse cross-finger flap of the long finger onto the index finger. The pedicled flap (4cm x 3cm) was flipped to cover the soft tissue of the index finger. We then harvested a 4cm x 3cm full-thickness skin graft from the ipsilateral upper arm and sutured it on top of the flap (Figure 3). Two weeks following her first operation, the patient underwent a successful second operation to divide the flap between the two digits. Following her second surgery, the patient was referred to occupational therapy where she was advised to follow a home exercise program to limit stiffness of the digits and hand. 

**Figure 4:**
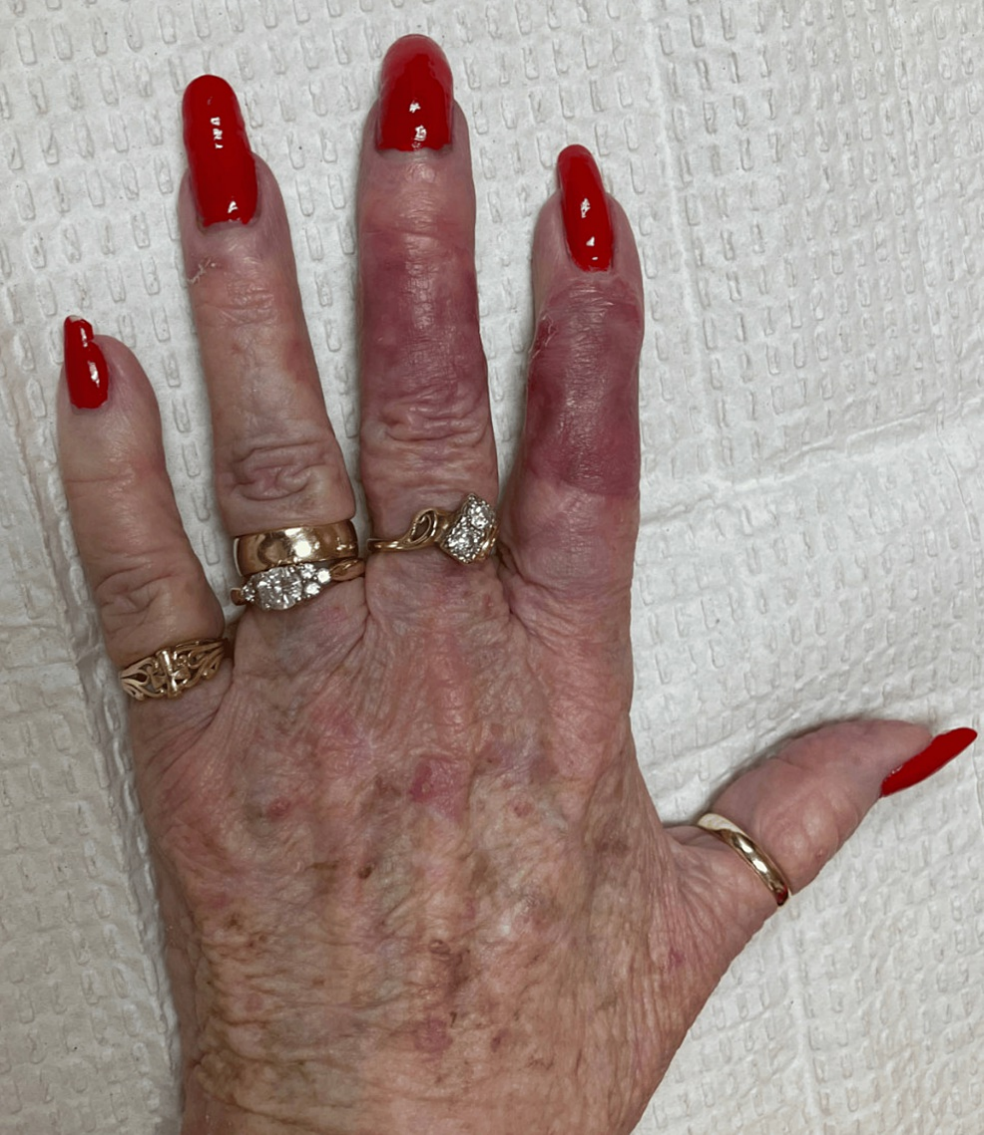
Patient’s wound eight weeks following surgery demonstrates exceptional healing.

Postoperatively, due to the excision of the extensor tendon, the distal interphalangeal joint demonstrated 20 degrees of extension lag. Nonetheless, the patient showed no other obvious defects or was able to make a loose composite fist. She was last seen eight weeks following her first operation and was satisfied with the appearance and function of her finger (Figure 4). 

## Discussion

BCC of the hand is a rare presentation in hand clinics and is even more infrequent when it presents on the digits. Due to its strong association with ultraviolet radiation from the sun, it is relatively more common to be found in areas of the body that receive more direct sunlight including the face and neck. However, one area that receives strong sun exposure yet presents with fewer cases of BCC is the dorsum of the hand. One such explanation for this occurrence is that BCC has been linked to originate from sebaceous glands, which are commonly found in the neck and face, but sparse in the dorsal hand [5]. While BCC of the hand is uncommon, BCC of the finger is even more infrequent. One systemic review of 120 cases of BCC of the hand found that 60.8% of cases occurred on the dorsal surface (excluding the digits) while only 19.2% occurred on the fingers and 12.5% on the thumb (6.7% were not specified). There was only one case of recurrence following treatment [6]. Notably, BCC has a favorable outlook as it rarely metastasizes and generally does not recur following resection. The risk for recurrence depends on the size of the tumor, histological type, and location. Of these 120 cases, 85 (63.4%) were treated with surgical excision, 4 (3%) were treated with MMS, 2 (1.5%) were amputated, and 1 (0.7%) was treated with electrodessication and curettage [6]. An even scarcer location for BCC is the nail unit, with few cases reported in English literature [7]. A meta-analysis of BCC of the hand found that in cases that involved the nail unit, 43% were treated with MMS, 31% with surgical excision, and 19% with amputation [8].

Traditionally, the two standard therapies for BCC include surgical excision and MMS. Of these two techniques, MMS has shown superior outcomes with regard to recurrence rates. A randomized trial of 408 primary and 204 recurrent facial BCCs comparing surgical excision versus MMS reported a 10-year recurrence rate of 4.4% for MMS and 12.2% for standard excision [9]. Although MMS might have favorable recurrence outcomes, it tends to be more costly and time-consuming, thus, it may be reserved for the treatment of high recurrence rate tumors [10]. While MMS and surgical excision are commonly employed, amputation of the digit is also utilized if the patient presents in an advanced disease state. A literature review of 14 cases of BCC on the finger found four instances where amputation was used to treat the patient [11]. Naturally, patients exhibit reluctance to undergo finger amputation due to aesthetic reasons and the loss of native functionality of the hand.

Historically, the majority of cases are treated with surgical excision or MMS. It is important to note that the treatment option that is chosen ultimately depends on the size and the location of the mass, the histology and patient-specific circumstances, the available resources at the physician’s disposal, and the physician’s preference. In our situation, the patient presented to us after she was seen by Dermatology and informed that surgical amputation might be a possibility. Desiring a different treatment route, she sought our opinion, and we offered her a reverse cross-finger flap of the long finger onto the index finger. While it was not ideal to excise the extensor tendon for our patient, the deep margin of the excised tumor was not clear and thus, it was medically necessary for removal. We believe that utilizing a reverse cross-finger flap was one of the options for our patient as it simulated native anatomy while minimizing the risk of recurrence and providing the functionality and aesthetics the patient desired. Additionally, local flaps utilize tissue from adjacent structures and thus minimize differences in the native anatomy of the skin that would otherwise be present if distant grafts were harvested. In the end, the patient was aesthetically satisfied with her outcome with the avoidance of potential amputation.

Further research is needed to investigate the recurrence and the long-term outcome for BCC treated with reverse cross-finger flaps. Nonetheless, the local flap of the hand could serve as another tool in the armamentarium for hand surgeons in the surgical management of BCC.

## Conclusions

BCC of the hand is a rare occurrence, particularly on the digits. While standard therapies like surgical excision and MMS remain primary options, the presented case demonstrates the successful utilization of a reverse cross-finger flap as a viable alternative to amputation, balancing aesthetic satisfaction and functional preservation. Although further research is needed to assess the long-term outcome, this case contributes to the evolving armamentarium of options available to hand surgeons in the surgical management of BCC.
